# Individualized drug screening in cholangiocarcinoma using organoid models and patient-derived tumor xenograft

**DOI:** 10.1186/s12885-025-15495-w

**Published:** 2025-12-31

**Authors:** Pinsheng Han, Liuyang Zhu, Wen Tong, Sen Liu, Yongdeng Xu, Libo Wang, Tianze Wang, Tianyu Zhao, Yu Miao, Hao Chi, Tao Cui, Ze Wang, Long Yang, Yamin Zhang

**Affiliations:** 1 School of Medicine, Nankai University, Tianjin, 300071 China; 2 State Key Laboratory of Druggability Evaluation and Systematic Translational Medicine, Tianjin Institute of Pharmaceutical Research, Tianjin, 300301 China; 3 Department of Hepatobiliary Surgery, School of Medicine, Tianjin First Central Hospital, Nankai University, Tianjin, 300384 China; 4 Shandong Cancer Hospital and Institute, Shandong First Medical University, Shandong Academy of Medical Sciences, Jinan, Shandong 250117 China; 5 Department of Pharmacology, Shenyang Pharmaceutical University, Shenyang, 110016 China; 6 First Central Clinical College of Tianjin Medical University, Tianjin, 300192 China; 7 Hefei Tianhui Biotechnology Co., Ltd, Hefei, 230000 China

**Keywords:** Organoids, Patient-derived tumor xenograft, Cholangiocarcinoma, Drug screening, Whole exome sequencing

## Abstract

**Introduction:**

Cholangiocarcinoma (CCA) is a highly aggressive biliary malignancy with a very poor prognosis. How to screen the optimal chemotherapy regimen is crucial for enhancing the prognosis of CCA patients. The study aims to develop patient-derived tumor organoid (PDO) models and patient-derived tumor xenograft (PDX) models of CCA to simulate clinical responses to chemotherapy.

**Methods:**

Tumor tissues were collected from patients undergoing surgical resection and subsequently utilized to establish PDO and PDX models. Hematoxylin-eosin (H&E), immunohistology (IHC), and immunofluorescence (IF) were conducted to analyze the biological characteristics of these PDXs and PDOs. Whole exome sequencing (WES) was performed to identify the mutation types of primary tumor, PDO, and PDX. Drug sensitivity assays were conducted utilizing PDO and PDX models to compare clinical treatment responses.

**Results:**

In this study, we successfully established 18 PDO (success rate, 56.3%) models and 21 PDX models (success rate, 65.6%) from 32 patients diagnosed with CCA. PDO and PDX preserved the mutational profiles characteristic of the primary tumor samples. The drug screening results from PDOs demonstrated a correlation with the actual clinical response to chemotherapy regimens, and these findings were further validated in PDX models.

**Conclusions:**

Our findings indicate that the integration of PDO and PDX models can successfully guide clinical treatment strategies, facilitating effective personalized therapy for CCA patients.

**Supplementary Information:**

The online version contains supplementary material available at 10.1186/s12885-025-15495-w.

## Introduction

Cholangiocarcinoma (CCA) is a highly aggressive biliary malignancy with an extremely poor prognosis [1]. CCA can develop in any part of the biliary tree and is categorized into intrahepatic CCA (iCCA), perihilar CCA (pCCA), or distal CCA (dCCA) subtypes based on its anatomical location [2]. The incidence and mortality of CCA, particularly iCCA, are rising swiftly, with most patients diagnosed at advanced stages [3]. While complete surgical resection offers the only chance for a potential cure, only approximately 20%-30% of patients present with resectable disease [4, 5]. Despite recent advancements in immunotherapy and targeted therapies improving patient outcomes to some extent, systemic chemotherapy continues to be the main palliative treatment for CCA [6, 7]. Common chemotherapeutic drugs for CCA include gemcitabine, cisplatin, oxaliplatin, 5-fluorouracil (5-FU), and capecitabine. However, the effectiveness of chemotherapy can vary significantly for individualized patients. Consequently, novel approaches are urgently needed to predict the efficacy of personalized treatment strategies.

Patient-derived tumor organoid (PDO) models are three-dimensional (3D) structured culture systems established by isolating and culturing patient primary tumor cells, and patient-derived tumor xenograft (PDX) models involve the implantation of patient tumor tissue into immunodeficient mice to create individualized tumor models. Both models faithfully recapitulate the biological characteristics of primary tumors, thereby paving the way for personalized precision medicine [8, 9].

Recent studies have demonstrated the promising utility of PDOs in predicting chemotherapy response and guiding personalized therapy for CCA [10, 11]. For instance, one retrospective study demonstrated that PDO-guided adjuvant chemotherapy was associated with improved recurrence-free survival in ICC patients [10]. Similarly, other research with established CCA PDOs confirmed that PDO drug responses correlated with clinical outcomes [11]. However, these prior studies primarily relied on PDO models alone. While PDOs offer advantages for high-throughput screening, they lack the complex in vivo microenvironment and systemic drug metabolism present in PDX models. To bridge this gap, our study establishes a complementary platform integrating both PDO and PDX models from CCA patients. We hypothesize that the combination of rapid PDO screening followed by rigorous in vivo validation in PDX models can provide a more robust and translatable strategy for personalized drug selection. Therefore, the aim of this study is not only to validate the predictive capacity of CCA PDOs but also to cross-verify these findings in PDX models, thereby offering a comprehensive preclinical workflow for optimizing chemotherapy regimens in CCA.

## Materials and methods

### Patient specimens

The Tianjin First Central Hospital approved patient tumor samples in this study (approval no. 2020N221KY). Tumor tissues were collected from CCA patients who underwent surgical resection in the hepatobiliary surgery department at Tianjin First Central Hospital. Informed patient consent was obtained before surgery. Postoperative clinical data of CCA patients, encompassing anatomical subtype, sex, age, CEA, CA199, tumor size, histological type, degree of differentiation, TNM stage, and morphological subtype were collected from the medical record system in Table S1.

### Laboratory animals

All animal studies received approval from the Animal Ethics Committee of Tianjin Tian Cheng New Drug Evaluation Co., Ltd (IACUC no. 2021072702). Six-week-old male BALB/c-nu (nude) mice (20 ± 2 g) were obtained from Beijing Vital River Laboratory Animal Technology Co., Ltd and housed in the SPF animal room at the Biotechnology Center of Tianjin Pharmaceutical Research Institute.

### Tissue processing

Fresh tumor tissues were stored in RPMI-1640 medium (Gibco) with 10% fetal bovine serum (FBS) (Gibco) at 4℃ and transferred to the laboratory within 24 h. Then the tissues were washed three times for 3 min each in cold phosphate-buffered saline (PBS) containing penicillin/streptomycin. A small fragment of each tumor specimen was embedded in paraffin for histology tests, while the remaining tissue samples were utilized to establish PDO and PDX models.

### CCA PDO models establishment

Tumor tissue is finely shredded and digested at 37 °C in 5 mL of advanced DMEM/F12 medium (Gibco) containing 4 mg/mL Collagenase Type IV (Gibco) for 30–60 min with gentle shaking. After digestion, the single-cell suspension was filtered through a 100-µm cell strainer and centrifuged at 300×g for 5 min. The single-cell suspension was treated with Red Blood Cell Lysis Buffer (Solarbio) for 5 min at 4℃ to remove red blood cells, followed by centrifugation at 300×g for 5 min. Following cell counting, the precipitate was resuspended in cold PBS, combined with Matrigel (Corning) to achieve a density of 20,000 cells per 35 µL, and promptly seeded into a 48-well culture plate. After Matrigel solidification at 37℃ for 15–30 min, 250 µl of organoid culture medium was added per well, and the organoids were incubated at 37℃ with 5% CO_2_. The composition of culture medium for CCA organoids was shown in Table S2 [12–14]. The culture medium was replaced every 3 days, and organoids were mechanically passaged every 1–3 weeks once their diameter reached 300–500 μm. After five passages of expansion, the CCA PDOs were utilized for subsequent experiments.

### CCA PDO models drug screening

During the passage of organoids, they were embedded in the 96-well plate, with each well containing 500 cells per 7 µl of Matrigel, followed by adding 150 µl medium to each well. The organoids were cultured for 72 h and then chemotherapy drugs were added to the culture medium for 5 days. Gemcitabine and 5-FU (all from MedChemExpress) were dissolved in DMSO, while cisplatin and oxaliplatin (all from MedChemExpress) were dissolved in sterile water. The final DMSO concentration was ≤ 0.1% and shown to be non-toxic to organoids. Each drug concentration gradient included three replicate wells to avoid bias. Gemcitabine concentrations were 25 µM, 10 µM, 1 µM, 0.1 µM, 0.01 µM, 0.001 µM, and 0 µM. The drug concentrations for 5-FU, cisplatin, and oxaliplatin were 50 µM, 25 µM, 5 µM, 1 µM, 0.1 µM, 0.01 µM, and 0 µM. Cell viability in each well was assessed using CellTiter-Glo 3D Reagent (Promega) following the kit’s standard procedures. Cell viability was normalized against the solvent control (0 µM, 100% viability) and background control (medium with Matrigel only, 0% viability). The normalized viability was calculated as: % Viability = (Luminescence_drug - Luminescence_background) /(Luminescence_control - Luminescence_background)×100%. Dose-response curves were plotted using logistic regression with variable slope, and the half-maximal inhibitory concentration (IC50) for each group was calculated with GraphPad Prism 9.0. The area under the curve (AUC) was calculated and normalized by dividing it by the maximum possible area across the analyzed drug concentration range. Organoid responses to each therapeutic agent were categorized into three subgroups: sensitive (lowest 33% AUC), resistant (highest 33% AUC), and intermediate sensitive (middle 34% AUC) [15, 16].

### CCA PDX models establishment and drug screening

Tumor tissues were sectioned into 2 × 2 × 2 mm³ pieces, with the removal of connective and necrotic tissues. The mice were anesthetized, fixed, and disinfected, and the tumor sections were directly subcutaneously implanted into the animal’s back. The initial transplanted tumor was recorded as the P1 generation, which was propagated to the P3 generation. For each passage, five nude mice were used for subcutaneous implantation. To ensure high engraftment efficiency, several optimized techniques were employed: (1) young, immunodeficient mice (6 weeks old) were selected as recipients; and (2) for the Passage 1 generation, bilateral implantation was performed in both armpits to increase the success rate. Passaging was initiated upon tumors reaching ~ 1500 mm³. The harvested tumors were then cut into 2 × 2 × 2 mm³ fragments for establishing subsequent generations (Passage 2, Passage 3) by subcutaneous implantation into new mice (*n* = 5 per passage).

When iCCA-P25 tumor volume reached 50–100 mm³, the nude mice were randomly assigned to three groups of five animals each: vehicle control group, gemcitabine group, and oxaliplatin group. The gemcitabine was dissolved in DMSO and further diluted with physiological saline to a final DMSO concentration of 2.5%. Oxaliplatin was dissolved directly in physiological saline. The nude mice were injected intraperitoneally with gemcitabine (50 mg/kg), oxaliplatin (10 mg/kg), or an equivalent volume of the corresponding vehicle twice a week.

Similarly, when dCCA-P27 tumor volume reached 50–100 mm³, the mice were assigned to three groups: vehicle control group, gemcitabine group, and 5-FU group. Gemcitabine (50 mg/kg) was prepared as described above. 5-FU was dissolved directly in physiological saline. The nude mice were injected with gemcitabine (50 mg/kg), 5-FU (30 mg/kg), or an equivalent volume of the corresponding vehicle twice a week.

All tumor volumes were measured using a tumor volume meter (Peira TM900) every three days. During the 4-week treatment period, no significant decrease in body weight was observed in the drug-treated mice, with their weights remaining stable between 22 ± 2 g. After 4 weeks of treatment with the drug, the mice were euthanized by cervical dislocation, and the tumors were removed to measure tumor volume and weight. Tumor tissues were collected for histological analyses.

### H&E and histopathology

CCA PDOs were submersed with 4% paraformaldehyde (Solarbio) and embedded in 3% agarose (Biosharp). Tumor tissues also were submersed in 4% paraformaldehyde. Following dehydration, paraffin-embedded samples were sectioned into 3 μm slices and subjected to hematoxylin-eosin (H&E) staining, immunohistology (IHC), and immunofluorescence (IF) according to standard protocols. IHC staining for CK7(ab181598, Abcam, 1:5000), CK19 (ab76539, Abcam, 1:1000), and EPCAM (66316-1-Ig, Proteintech, 1:1000) was performed for patients’ tumor tissues and PDXs. IF staining for CK7(ab181598, Abcam, 1:100), CK19 (ab76539, Abcam, 1:100), and EPCAM (66316-1-Ig, Proteintech, 1:200) was performed for PDOs. Furthermore, IHC staining for Ki67 (27309-1-AP, Proteintech, 1:4000), CEA (68377-1-Ig, Proteintech, 1:2500), and P53 (60283-2-Ig, Proteintech, 1:2500) was performed for patients’ tumor tissues, PDXs, and PDOs.

### Whole-Exome Sequencing (WES)

According to the manufacturer’s protocol, DNA was extracted from PDOs, PDXs, and corresponding tumor tissues using the Magnetic Universal Genomic DNA Kit (Tiangen). WES data were analyzed, and the variants were identified through the following steps: First, quality control of the raw reads was performed using Fastp (v0.21.0), and low-quality reads were removed. For PDX samples, mouse-derived reads were filtered out utilizing Xenome (v1.0.0). The cleaned reads were subsequently aligned to the UCSC hg38 reference genome using Bowtie2 (v2.2.9). PCR duplicates were removed from the aligned BAM files using Picard (v2.25.0) with the “AddOrReplaceReadGroups” and “MarkDuplicates” tools, and local realignment was performed using GATK (v3.8.0) with the “RealignerTargetCreator” and “IndelRealigner” tools. Variant calling was carried out using VarScan (v2.3.9), and copy number variations (CNVs) were detected with the R package cn.mops (v1.48.0). Variant annotation was first performed with snpEff (v2024-04-09) to filter out non-relevant sites, such as intergenic and intronic regions. Further annotation was done using Annovar (v2020-06-08) databases including dbSNP, COSMIC (v100), ExAC, and SIFT. To identify cancer-associated variants, the following filtering criteria were applied: first, SNPs present in the dbSNP database were removed, followed by filtering out variants with a frequency > 0.01 in ExAC. Synonymous and intronic variants were also excluded. Variants present in COSMIC were retained, and mutations predicted by SIFT to have a potentially deleterious effect on the corresponding encoded protein (SIFT score < 0.05) were selected.

### Fluorescein TUNEL staining

The apoptosis level of the PDX with treatment was detected using a TUNEL In Situ Apoptosis Kit (Elabscience). Following dewaxing, slides were incubated with Proteinase K for 15 min at 37 °C, then treated with TUNEL-FITC in dark for 60 min. DAPI was incubated for 5 min in the dark. The images were observed and collected under the fluorescence microscope.

### Statistical analysis

All statistical analyses were performed using GraphPad Prism software (GraphPad 9.0). Continuous data from PDX efficacy studies (including tumor volume, tumor weight, Ki67 positivity rate and TUNEL positivity rate) are presented as mean ± standard deviation (SD), as they approximated a normal distribution. Conversely, the PDX tumor formation time, which represented time-to-event metrics and showed a non-normal distribution, are summarized using median and range. To compare the differences among multiple groups in efficacy studies, one-way analysis of variance (ANOVA) was used. Following a significant ANOVA result (*p* < 0.05), Tukey’s honestly significant difference (HSD) post-hoc test was applied for all pairwise comparisons between groups. A *p*-value of less than 0.05 was considered statistically significant.

## Results

### Establishment of PDX and PDO models from CCA patient tissue

**Fig. 1:**
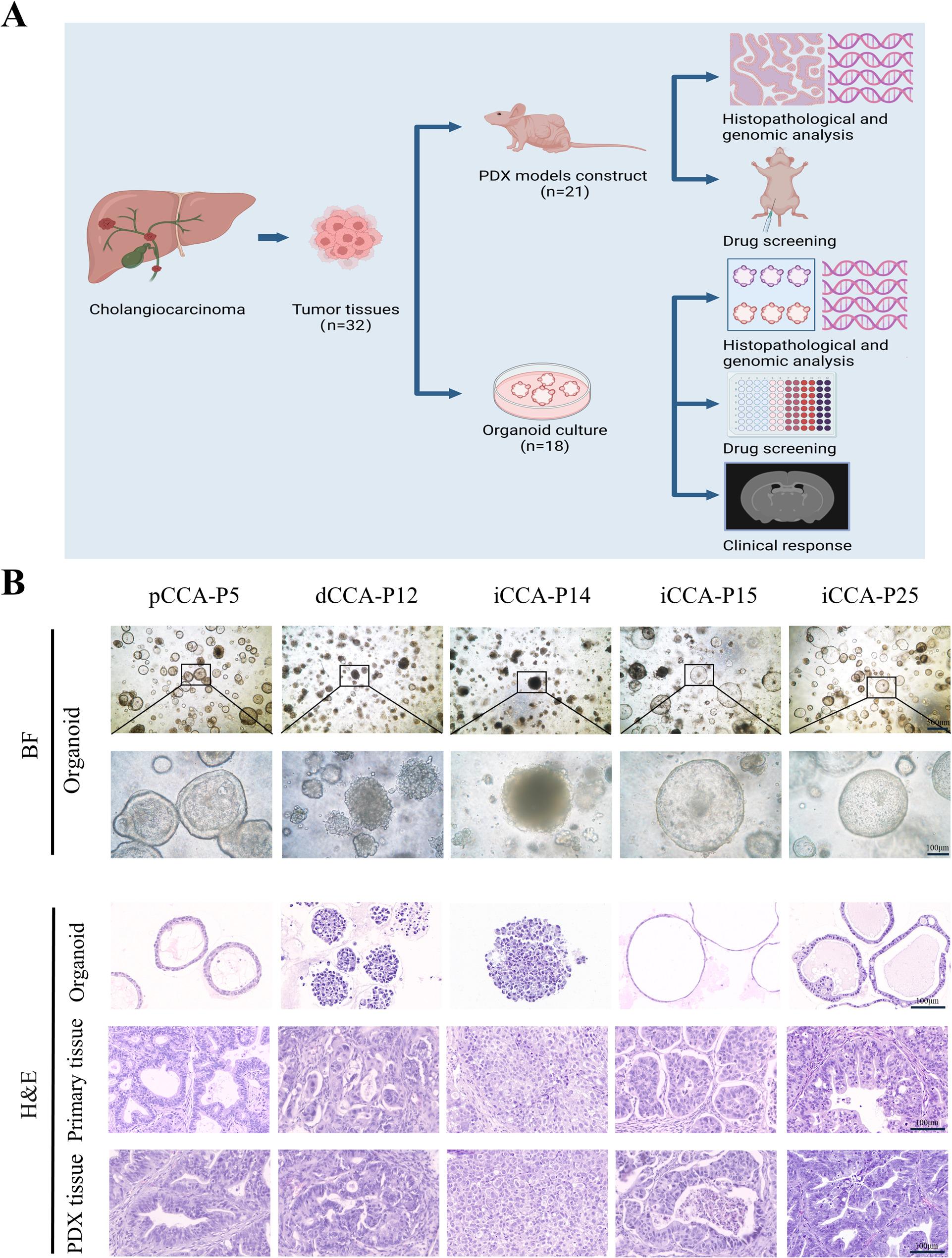
Establishment of CCA PDX and PDO models. **A** Flow diagram of the study, including the establishment of CCA PDXs and PDOs, histological characterization, genomic analysis, and drug screening in PDXs and PDOs. **B** Histopathological features of primary tumors, PDXs, and PDOs. Bright-field and H&E comparison of five CCA PDXs and PDOs with the corresponding tumor tissue. Scale bar, 100 μm or 500 μm. iCCA, intrahepatic cholangiocarcinoma; pCCA, perihilar cholangiocarcinoma; dCCA, distal cholangiocarcinoma; PDX, patient-derived tumor xenograft; PDOs, patient-derived tumor organoids

We established CCA PDX and PDO models using 32 tumor samples from CCA patients who underwent surgical resection at our center (Fig. 1A). 21 PDXs were successfully cultured (success rate, 65.6%), including 14 iCCA PDXs, 5 dCCA PDXs, and 2 pCCA PDXs, consistent with prior research [17]. Additionally,18 organoids were successfully cultured (success rate, 56.3%), comprising 12 iCCA organoids, 4 dCCA organoids, and 2 pCCA organoids, consistent with previous research [18, 19]. The predominant pathological type was adenocarcinoma, with the majority of tumors exhibiting moderate to poor differentiation. To further characterize the PDX models, we analyzed their establishment timeline. The PDX models were serially passaged to P3, and the median time for initial engraftment (Passage 1) was 49 days (range: 27-109 days). The growth accelerated upon passaging, with median times shortening to 39 days (Passage 2) and 31 days (Passage 3), demonstrating successful in vivo adaptation (Table S3). We further assessed the stability and growth characteristics of the PDX models across passages. The tumor take rate increased to 100% in subsequent passages (Passage 2 and Passage 3), demonstrating high consistency once a model was established. However, we observed considerable variability in growth kinetics. The tumor formation time for the Passage 3 varied widely from 18 to 70 days (Table S3), indicating heterogeneous growth rates among the different PDX lines that mirrors the inter-tumoral diversity of CCA. Similarly, the PDO models were stably passaged to Passage 10. All these PDXs and PDOs were cryopreserved and successfully thawed for regeneration. 

### PDX and PDO models maintained histological features similar to those of the primary tumors

**Fig. 2:**
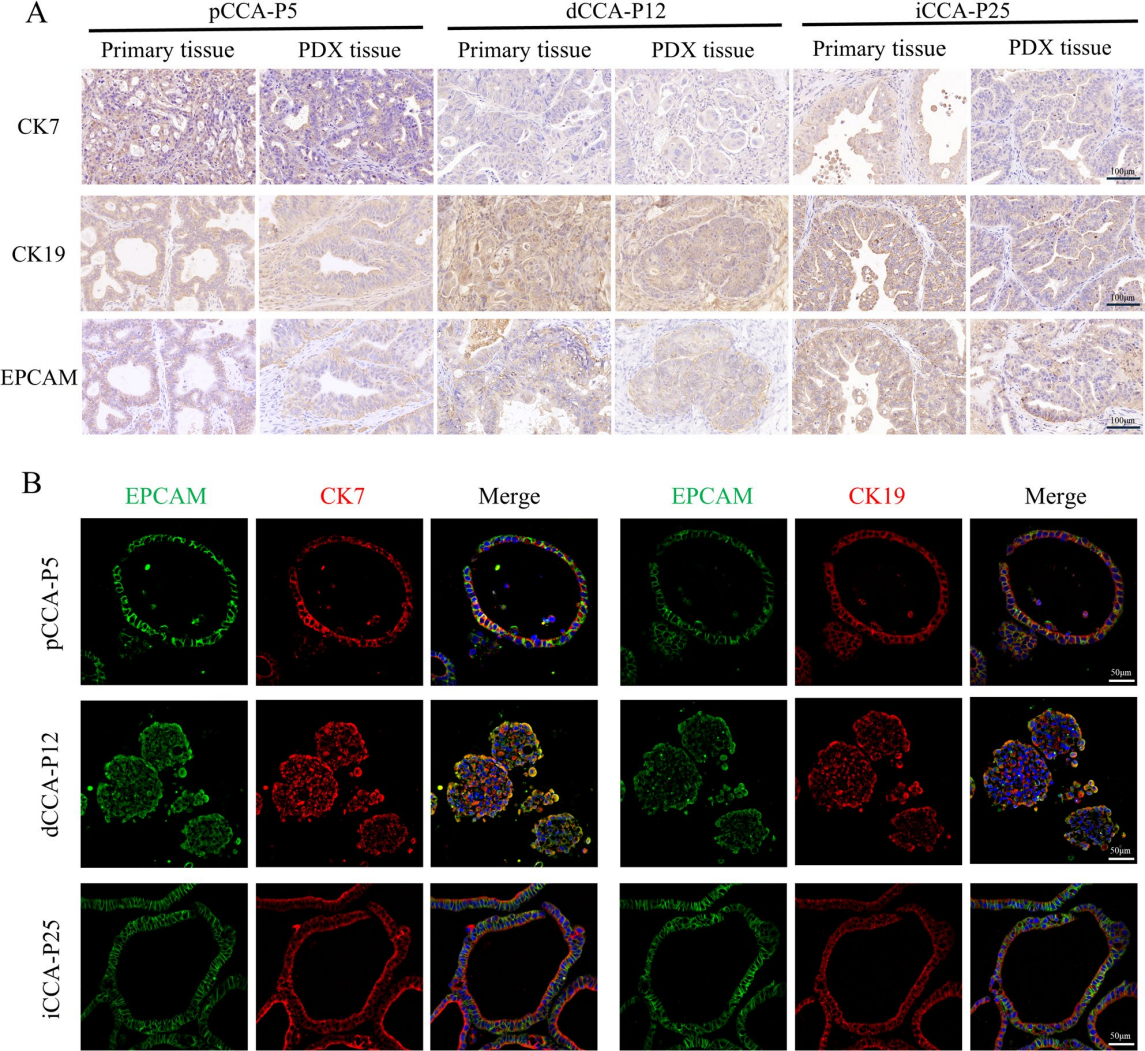
Bile duct marker expression analysis of CCA PDXs and PDOs. The expression of biliary markers (CK7, CK19, and EPCAM) was evaluated in CCA PDXs and PDOs, and compared against their matched primary tumors. **A** IHC staining for primary tumor tissues and PDXs. **B** IF staining for PDOs. Scale bar, 50–100 μm

Organoids exhibited a range of morphologies, such as cystic thick wall type, cystic thin wall type, and circular compact type (Fig. 1B). The histological characteristics of CCA PDXs and PDOs were further assessed. We confirmed that CCA PDXs and PDOs preserved histological characteristics akin to their originating primary tumors (Fig. 1B). The PDXs and PDOs protein expression pattern of bile duct markers, including CK7, CK19, and EPCAM, was consistent with original tissue samples (Fig. 2).

**Fig. 3:**
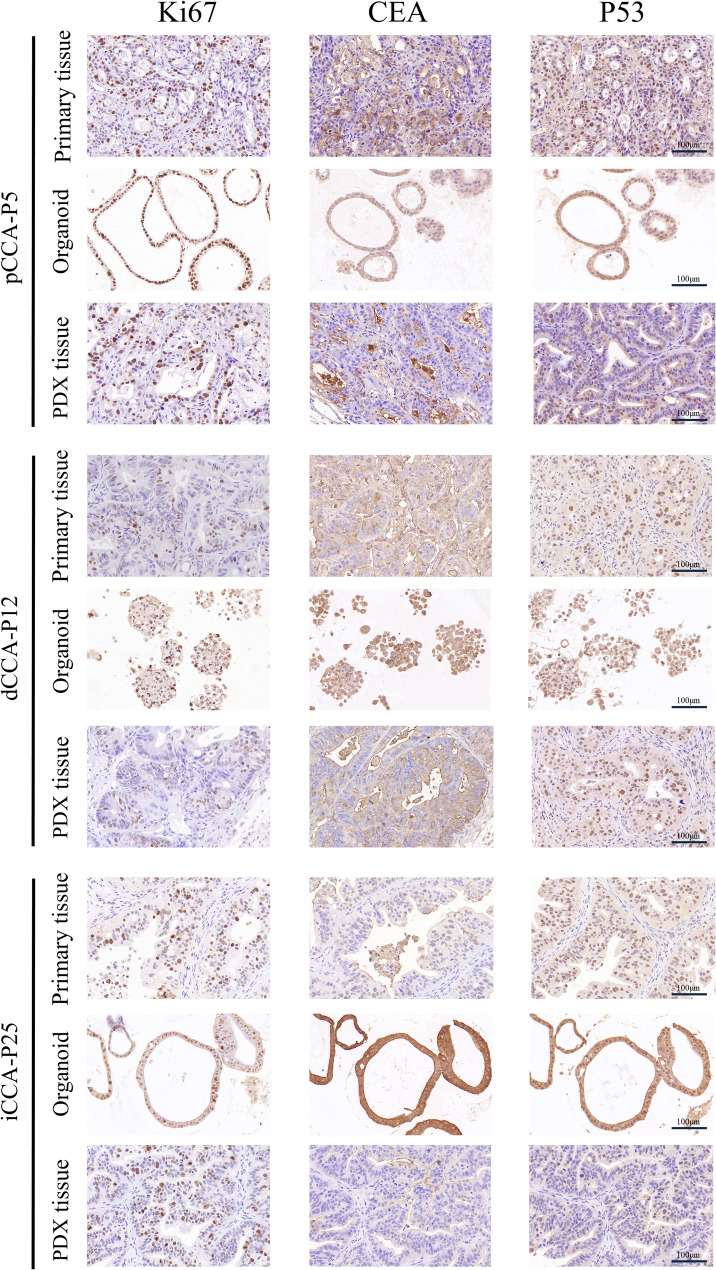
Tumor signature molecules expression analysis of CCA PDXs and PDOs. CCA PDXs and PDOs are compared to their primary tumors for tumor signature molecule Ki67, CEA, and P53 IHC staining. Scale bar, 100 μm

The expression of Ki67, CEA, and P53 was analyzed as they represent key biomarkers with prognostic and therapeutic relevance in CCA. Ki67 is a proliferation marker whose expression level is closely associated with aggressive tumor behavior and poor prognosis in CCA. CEA is a widely used clinical serum tumor marker for CCA, and its expression can correlate with disease progression. P53 tumor suppressor protein is frequently mutated in CCA, and aberrant accumulation (as detected by IHC) is a marker of dysfunction and poor prognosis. The expression analysis of these tumor signature molecules demonstrated that PDXs and PDOs maintained analogous staining patterns corresponding to primary tumors for Ki67, CEA, and P53 (Fig. 3). These proteins are considered potential biomarkers for predicting the prognosis of CCA. In brief, we successfully established CCA PDX and PDO models that preserved the histological characteristics of the primary tumors.

### Genomic characterizations of PDOs and PDXs

**Fig. 4:**
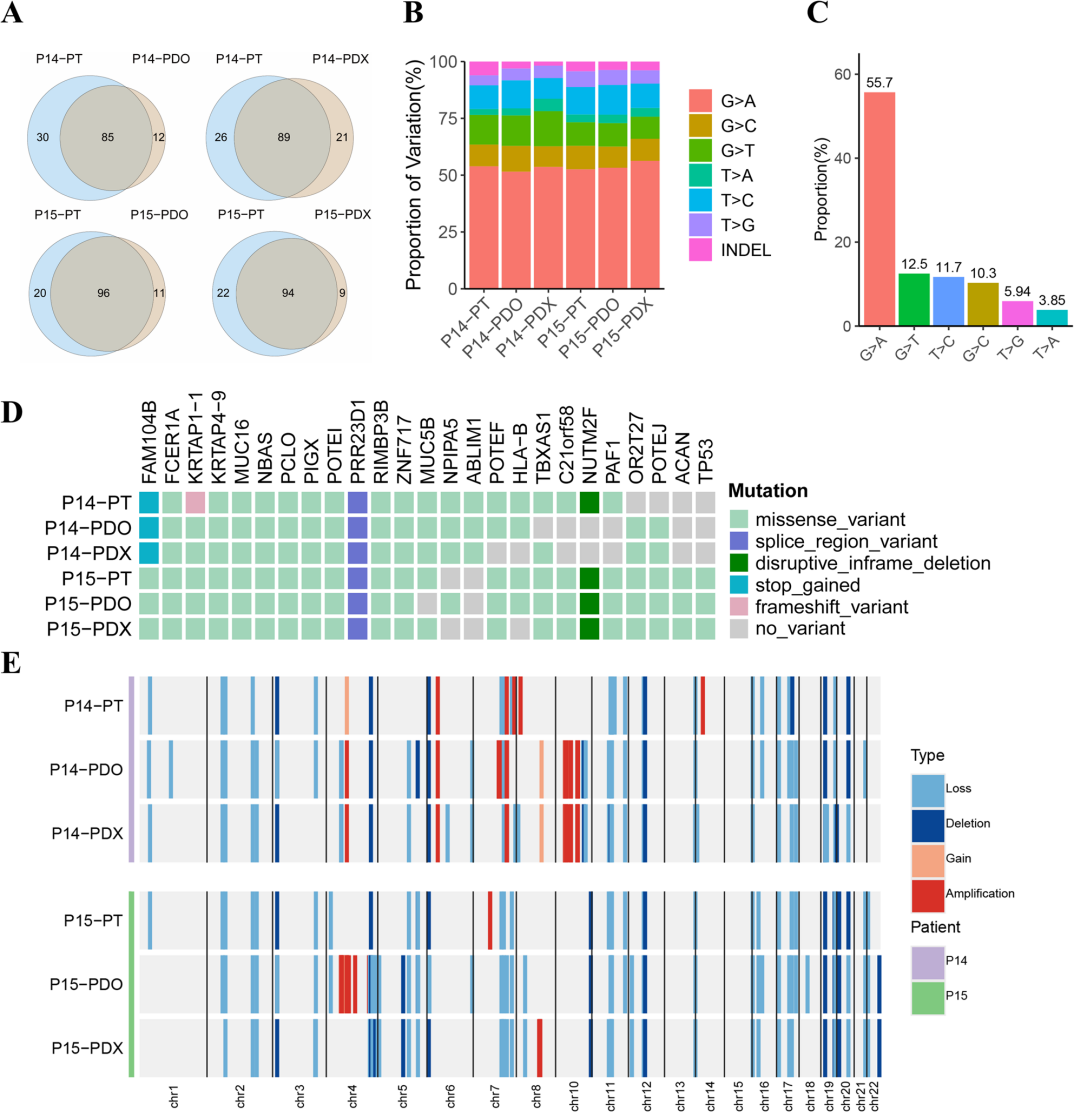
Genomic landscape of CCA PDOs, PDXs, and corresponding primary tumor. **A** Venn diagrams showing the number of cancer-associated non-synonymous mutations in two CCA PDOs, PDXs, and their corresponding primary tumor tissues. **B** The Distribution of each exonic variant across the samples, including the six types of single nucleotide variants (SNVs), and insertions and deletions (Indels). **C** The average percentage of the six types of SNVs across all samples is presented. **D** A landscape map of the top 25 cancer-associated mutations in CCA, with mutation types indicated in the legend. **E** Copy-number analysis in matched primary tumor tissues and corresponding PDOs, PDXs. PT: Primary tumor

To assess whether the PDOs and PDXs retain the mutational characterizations of their corresponding primary tumors, we performed the WES analysis on PDOs, PDXs, and primary tumors from patient iCCA P14 and iCCA P15 (Fig. 4). Both primary tumors harbored approximately 100 tumor-related mutations, the majority of which were reserved in the PDOs and PDXs (Fig. 4A). The proportions of exonic variations in the primary tumors were similarly maintained in PDOs and PDXs (Fig. 4B). In addition, the distribution of base substitutions showed that G > A mutations were the pre-dominant variants in both primary tumors and corresponding cultures (Fig. 4C). Significantly, both the tumor-related mutations (Fig. 4D and Table S4) and CNVs (Fig. 4E) in the PDOs and PDXs accurately reflected the genomic alterations in CCA. Additionally, according to Fig. 4D, we identified 12 genes (FAM104B, FCER1A, KRTAP1-1, KRTAP4-9, MUC16, NBAS, PCLO, PIGX, POTEI, PRR23D1, RIMBP3B, ZNF717) harboring mutations that were consistently shared between the primary tumor, PDO, and PDX models in both patients P14 and P15. Notably, several of these genes have established or emerging roles in human cancers. For instance, MUC16 (encoding CA-125) is highly expressed and associated with poor prognosis in CCA [20]. The PCLO mutation has been reported as a predictive biomarker for poor overall survival in oral squamous cell carcinoma [21]. Additionally, mutations in PIGX have been identified in lung adenocarcinomas from young never-smokers [22], and ZNF717 mutations are present in colorectal cancer [23]. The conservation of these specific mutations in our models highlights their potential relevance to CCA pathogenesis and warrants further investigation.

### Drug screening to chemotherapeutics of CCA PDOs

Gemcitabine, 5-FU, cisplatin, and oxaliplatin have been the most common first-line chemotherapeutic agents for CCA treatment in recent decades. To investigate the responses of CCA PDOs to various chemotherapeutic agents, four drugs were administered to 18 CCA organoids. Four drugs were evaluated at six distinct concentrations across three replicate wells respectively. Viable cell ratios were quantified post-treatment for each group to generate dose-response curves and determine IC50 and AUC (Tables S5 and S6). Organoids for each therapeutic agent were categorized into three subgroups: sensitive (lowest 33% AUC), resistant (top 33% AUC), and intermediate response (middle 34% AUC).

**Fig. 5:**
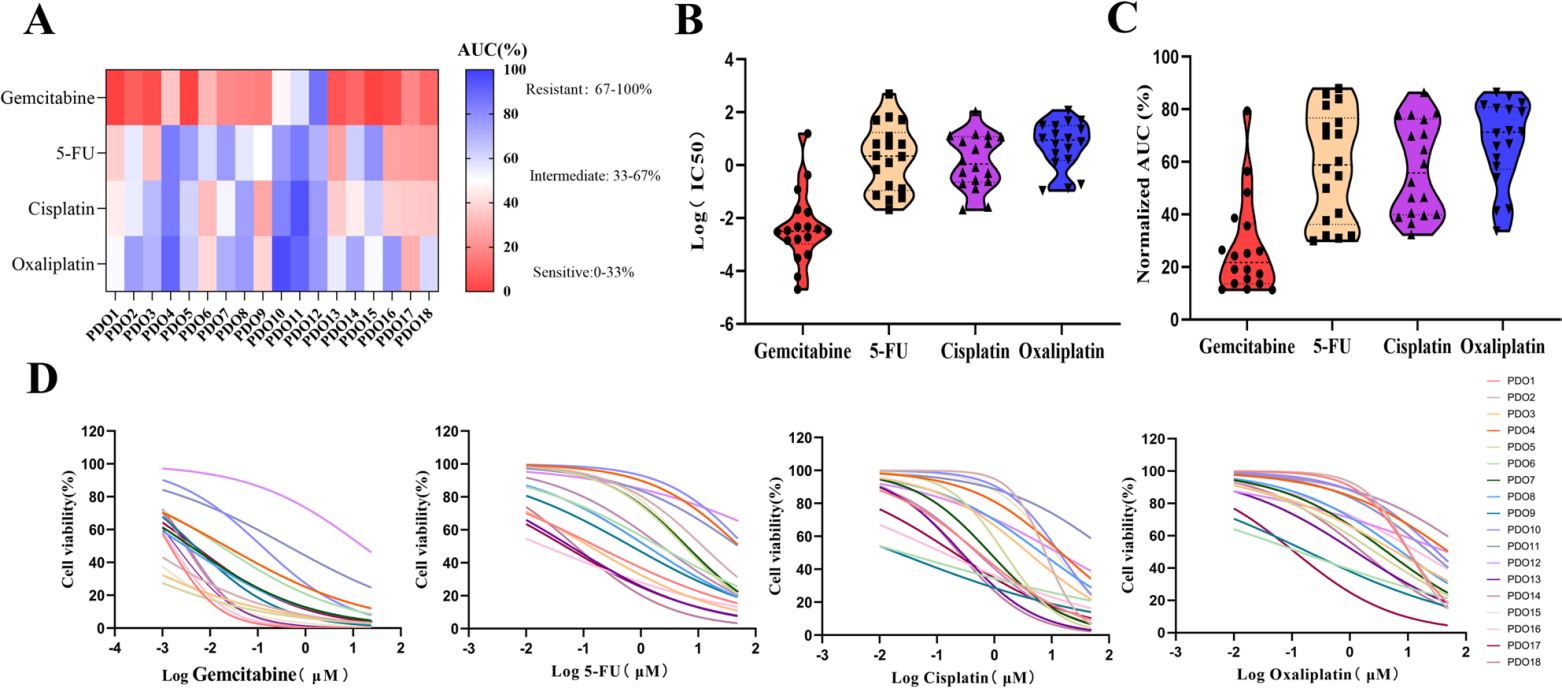
Drug-screening for chemotherapy in CCA organoids in vitro. **A** The heatmap consists of 18 CCA organoids’ chemotherapy sensitivity responses, which included gemcitabine, 5-FU, cisplatin, and oxaliplatin (n = 3). Chemotherapy sensitivity responses were evaluated by AUC%. Colors range from blue (high AUC%, meaning resistant) to red (low AUC%, meaning sensitive). **B** IC50 was calculated derived from the raw dose-response data and was presented using a violin plot. **C **AUC was calculated derived from the raw dose-response data and was presented using a violin plot. **D** Summary of chemotherapy responses for 18 CCA organoids and results were illustrated through dose-response curves. IC50, the half maximal inhibitory concentration; AUC, the area under the curve

Responses to chemotherapeutic drugs, including gemcitabine, 5-FU, cisplatin, and oxaliplatin, showed significant variation among organoids derived from different CCA patients (Fig. 5A). CCA PDOs exhibited notable interpatient differences in their sensitivity to individual chemotherapy drugs according to IC50 values and AUC values (Fig. 5B and C). Some organoids demonstrated resistance, while others exhibited intermediate sensitivity or sensitivity to the above common chemotherapy drugs as determined by dose-response curves (Fig. 5D). If PDOs are sensitive to chemotherapy, they exhibit cell death and destruction of 3D structures, while when PDOs are resistant to chemotherapy, they can still maintain their own 3D structures compared with the control group. The organoid-formation assay revealed distinct drug sensitivity profiles among the PDO lines (Figure S1). Specifically, pCCA-PDO2 was sensitive to gemcitabine but resistant to 5-FU, cisplatin, and oxaliplatin. iCCA-PDO7 exhibited sensitivity to gemcitabine, intermediate sensitivity to cisplatin, and resistance to 5-FU and oxaliplatin. In contrast, iCCA-PDO12 was resistant to all four agents tested. pCCA-PDO13 was sensitive to gemcitabine and 5-FU, and showed intermediate sensitivity to cisplatin and oxaliplatin. Finally, iCCA-PDO14 was sensitive to gemcitabine, exhibited intermediate sensitivity to 5-FU and cisplatin, and was resistant to oxaliplatin. These findings indicate that CCA PDOs are a promising platform for assessing the drug responses to conventional chemotherapeutics.

### Drug response status observed in CCA PDOs was validated in PDX models

**Fig. 6:**
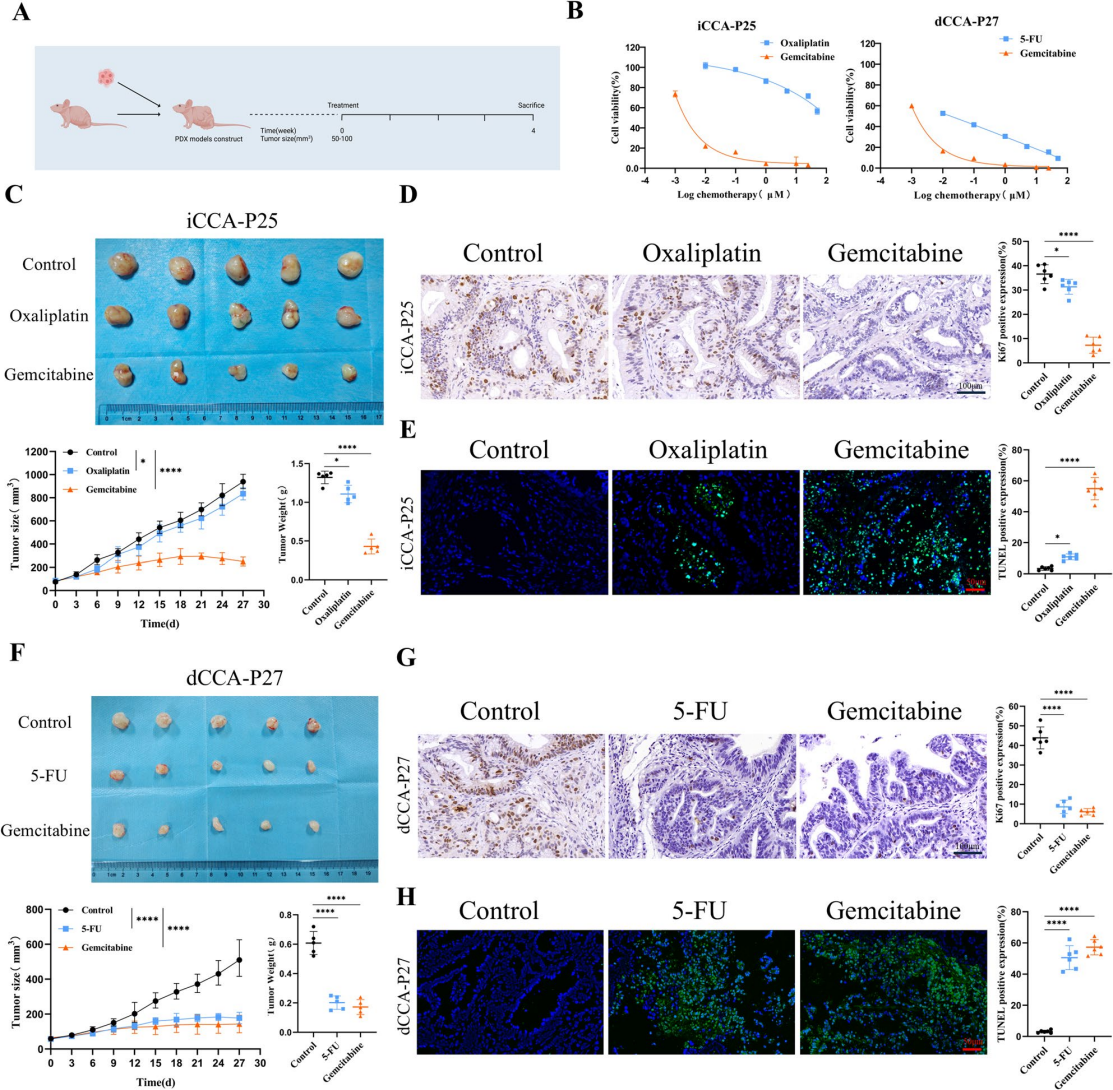
Drug response status in CCA PDOs were validated in the PDX model. **A** Experimental design. PDX tumors were subcutaneously transplanted into nude mice and administered either the vehicle or chemotherapeutic drugs twice a week when tumor volumes reached 50–100 mm^3^. After 4 weeks of treatment with the drug, the nude mice were euthanized, and the tumors were removed and weighed. **B** ICCA-P25 and dCCA-P27 PDO drug sensitivity profiles. **C** Representative image and growth curves of iCCA-P25 PDX (*n* = 5). **D** Representative immunohistochemistry (IHC) images of Ki-67 and the following quantification of Ki-67-positive cells in iCCA-P25 PDX (n = 6). Scale bar, 100 μm. **E** Representative TUNEL staining images and corresponding quantification of the relative intensity of TUNEL-positive in iCCA-P25 PDX (n = 6). Scale bar, 50 μm. **F** Representative image and growth curves of dCCA-P27 PDX (*n* = 5). **G** Representative IHC images of Ki-67 and the following quantification of Ki-67-positive cells in dCCA-P27 PDX (n = 6). Scale bar, 100 μm. **H** Representative TUNEL staining images and corresponding quantification of the relative intensity of TUNEL-positive in dCCA-P27 PDX (n = 6). Scale bar, 50 μm. Results represent mean ± SD, * *P* < 0.05, **** *P* < 0.0001. One-way ANOVA was used to compare between different groups

PDX was utilized to verify drug response for corresponding CCA PDOs. The iCCA-P25 and dCCA-P27 models were selected for PDX drug validation based on their representation of key anatomical CCA subtypes and the discriminative drug response profiles (including both sensitive and resistant responses) observed in their corresponding PDO screenings. When the iCCA-P25 or dCCA-P27 transplanted tumors grew to a size of 50–100 mm³, mice with subcutaneous tumors of similar size were randomly divided into groups to receive either chemotherapeutic drugs or a control treatment over a period of 4 week. (Fig. 6A). The iCCA-P25 PDO exhibited sensitivity to gemcitabine and resistance to oxaliplatin, while dCCA-P27 PDO showed sensitivity to gemcitabine and 5-FU (Fig. 6B).

Mice engrafted with iCCA-P25 PDX showed a significant response to gemcitabine treatment (*p* < 0.0001), with marked reductions in both tumor growth and tumor weight compared to untreated controls (Fig. 6C). Nevertheless, the iCCA-P25 PDX mice exhibited less tumor growth inhibition in response to oxaliplatin treatment, aligning with the findings for iCCA-P25 PDOs (Fig. 6B). Gemcitabine-treated iCCA-P25 PDX tumor tissues showed a significant decrease in cell proliferation (*p* < 0.0001) (Fig. 6D) and a huge increase in cell apoptosis (*p* < 0.0001) (Fig. 6E) compared to untreated PDXs and less difference was observed in the oxaliplatin-treated PDXs. Mice engrafted with dCCA-P27 PDX showed a significant response to gemcitabine (*p* < 0.0001) and 5-FU (*p* < 0.0001) (Fig. 6F), consistent with the discovery of dCCA-P27 PDO (Fig. 6B). Meanwhile, gemcitabine and 5-FU treatment dCCA-P27 PDX tumor tissues showed a significant decrease in cell proliferation (*p* < 0.0001) (Fig. 6G) and a huge increase in cell apoptosis (*p* < 0.0001) (Fig. 6H).

These findings collectively confirm that the drug screening results obtained from CCA PDOs were validated in the PDX models in vivo.

### Correlation between CCA PDOs drug sensitivity and clinical treatment responses

**Fig. 7:**
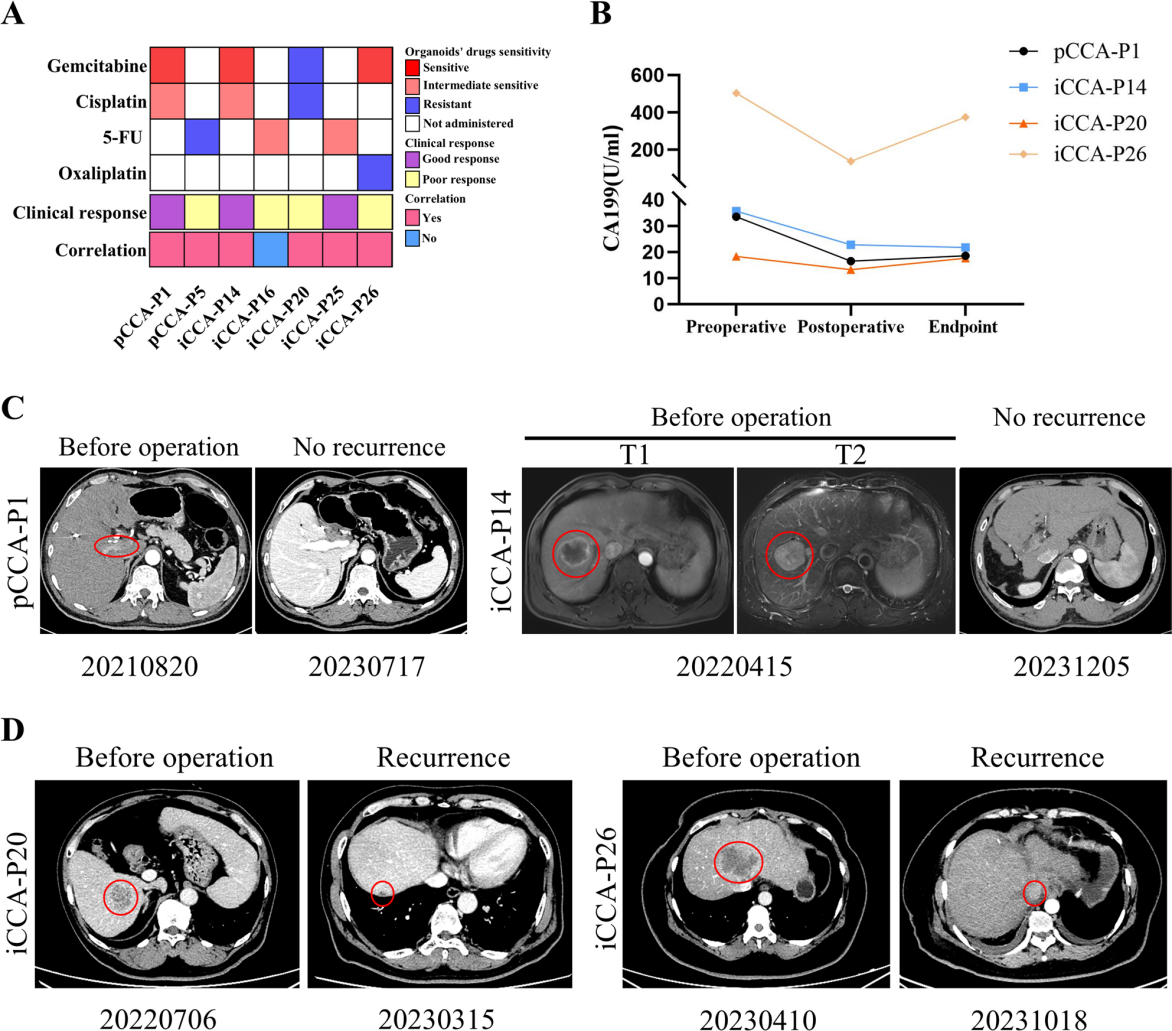
Comparison of drug response between CCA patients on chemotherapy treatment and their corresponding cancer organoids. **A** The heatmap summarizes the results for the 7 CCA PDOs and corresponding patient drug responses. Comprehensive details on the specific chemotherapy regimens received by all patient cohorts are provided in Table S7. **B** Serum CA199 levels in CCA patients correlate with clinical response and PDO predictions. Endpoint represent recurrence or disease-free survival > 17.5 months. **C-D** The imaging manifestations of target lesions in 4 CCA patients who received adjuvant chemotherapy following surgery, including tumor no recurrence in patients pCCA-P1 and iCCA-P14, as well as recurrence in patients iCCA-P20 and iCCA-P26.

Previous studies have demonstrated that the results of drug screening tests conducted on PDOs correlated with the patient clinical responses to chemotherapy [11]. We collected clinical data from seven CCA patients who received adjuvant chemotherapy after surgery (Table S7). The BILCAP trial [24, 25] defines patients who experience recurrence within 17.5 months after surgery as clinical poor responses to chemotherapy. Among these patients, patients pCCA-P1, iCCA-P14, and iCCA-P25 were clinically sensitive responders, while patients pCCA-P5, iCCA-P16, iCCA-P20, and iCCA-P26 were clinically resistant responders (Fig. 7A). According to drug screening results in CCA PDOs, we defined the chemotherapy regimen as resistant when it contained a resistant chemotherapeutic drug [15]. Six of the seven cases exhibited consistency between CCA PDO drug sensitivity results and the clinical response of the corresponding patient.

To further obtain longitudinal and objective biochemical evidence of treatment response, we monitored the serum levels of CA199, a cornerstone clinical tumor marker for CCA, in these patients. We analyzed CA199 levels at three time points: preoperative, postoperative, and at the follow-up endpoint (recurrence or disease-free survival > 17.5 months). As shown in Fig. 7B, distinct trajectories were observed between clinically sensitive and resistant patients. In the sensitive patients (pCCA-P1 and iCCA-P14), CA199 levels dropped significantly after surgery and were maintained at low levels throughout the extended disease-free follow-up period. In contrast, in the resistant patient iCCA-P26, CA199 levels rebounded dramatically at the time of tumor recurrence, providing a clear serological correlate of disease progression. The resistant patient iCCA-P20 also showed an elevated CA199 level at recurrence, albeit to a lesser extent. These dynamic CA199 profiles reinforce the clinical response classifications and align with the drug sensitivity predictions from our PDO models.

Patients pCCA-P1 and iCCA-P14 remained disease-free survival for more than 19 months after receiving GP (gemcitabine and cisplatin) chemotherapy regimens after surgery and were clinically sensitive patients to GP chemotherapy (Fig. 7C). These two PDOs showed a sensitive response to gemcitabine and an intermediate sensitive response to cisplatin. Patient iCCA-P20 received the 4 times GP chemotherapy regimen after surgery, but the tumor recurred after 8 months postoperatively (Fig. 7D), meanwhile whose PDO displayed a resistant response to gemcitabine and cisplatin. Similarly, Patient iCCA-P26 received 4 times GEMOX (gemcitabine and oxaliplatin) chemotherapy regimens after surgery, but the tumor occurred recurrence after 6 months postoperatively (Fig. 7D), whose PDO showed a resistant response to oxaliplatin.

The CCA PDO drug sensitivity test results in vitro accurately reflect the clinical response to chemotherapy regimens in corresponding patients. Consequently, the organoid model holds significant potential for assisting in the selection of appropriate clinical chemotherapy regimens.

## Discussion

Despite the growing number of therapy regimens, including chemotherapy, targeted therapy, and immunotherapy, the prognosis and treatment response for CCA patients remain unsatisfactory [26]. Effective individualized drug choices for CCA patients are critically important. PDO and PDX models accurately maintain the primary tumor’s biological characteristics and gene heterogeneity, showing distinct benefits for personalized treatment and predicting patient responses to therapy [27–29].Unfortunately, establishing a PDX model is often time-consuming, and provides little benefit for patients with rapidly progressing diseases. Although establishing a PDO model is time-efficient, its application in drug testing is limited due to the absence of extracellular matrix, immune cells, and functional vascular [30]. This study screened common chemotherapy regimens using both PDO and PDX models. The PDO models were used for initial large-scale drug screening, whereas the PDX models were employed to validate drug efficacy, rendering the two models complementary.

PDX models are established by implanting segmented tumor fragments from CCA patients into immunodeficient mice, subcutaneously or orthotopically. Our study confirmed that PDX models retain the histological characteristics of the primary tumor and the heterogeneity of cancer cells, as demonstrated by IHC and IF (Figs. 2 and 3). However, establishing PDX models is fraught with challenges, with success rates varying depending on multiple factors such as tumor type, implantation site, recipient mouse strain, and the interval between surgical resection and transplantation [31]. Compared to other malignancies, CCA exhibits an elevated degree of malignancy, leading to an increased success rate. In this study, the success rate in CCA PDX models was approximately 65.5% (21/32), aligning with a previous report of 55.1% (49/89) [17]. The transplantation site has been reported to significantly influence xenograft tumor growth [32]. Subcutaneous transplantation of PDX models is commonly used attribute to its simplicity and ease of tumor size measurement. Orthotopic models are anticipated to more precisely replicate tumor behavior, particularly in metastasis [33], but personnel with advanced surgical skills are needed to generate appropriate PDX models. Therefore, given the limitations of the technique, subcutaneous transplantation is more appropriate for large-scale research. In this experiment, nude mice were utilized to establish CCA PDX models, which are characterized by the absence of a functional thymus and T lymphocytes. At present, the most successful application of PDX model construction is observed in NOG or NSG mice, which not only retain the characteristics of SCID mice lacking T and B lymphocytes but also exhibit significantly reduced activity of their innate immune system, including the complement system and natural killer (NK) cells [34, 35]. Our preliminary experiments found that the success rates in nude mice and NOG or NSG mice were similar. Therefore, considering cost-effectiveness, we chose nude mice to construct CCA PDX models. However, CCA PDX models are both costly and time-consuming with a long engraftment period, rendering them unsuitable for large-scale drug screening applications.

In our study, we successfully established 18 PDOs with a success rate of 56.3% from 32 surgically excised samples of patients with CCA. While PDXs have a slightly higher success rate than PDOs, new culture methods are being developed to improve the success rate of organoid generation [36]. The application of various growth factors that promote the stemness of CCA cells holds the potential to improve the success rate of PDOs [37].

PDO models have been extensively utilized for large-scale drug screening and conducting preclinical trials to assess drug efficacy [38–40]. In this study, we conducted in vitro drug screening using PDOs to compare with clinical treatment response. Capecitabine, GEMOX, and GP regimens are considered the most suitable adjuvant chemotherapy options for CCA patients after surgery, although clinical responses to these treatments exhibit considerable variability among these patients. Our findings revealed significant heterogeneity in drug responses among CCA organoids, consistent with the diverse clinical responses with these regimens. This finding indicates that the PDO model can potentially serve as a valuable preclinical tool for drug screening.

Capecitabine, an oral fluoropyrimidine chemotherapy agent, is converted to 5-FU within liver and tumor tissues through the three-step enzymatic reaction. Tumor tissues exhibit high levels of thymidine phosphorylase, an enzyme integral to 5-FU metabolism, thereby contributing enrichment of 5-FU in tumors. Capecitabine has shown tumor suppressive efficacy across various PDX models [41, 42]. Given that the PDO model lacks an effective metabolic pathway, we switched from capecitabine to 5-FU for drug testing, highlighting a limitation in the drug-screening process of the PDO. Notably, researchers have developed an integrated biomimetic array organoid chip, where "liver tissue" metabolizes capecitabine into 5-FU to eradicate tumor cells [43].

Immunotherapy represents a transformative approach in oncology, aimed at activating the systemic immune response and alleviating immunosuppression within the tumor microenvironment [44, 45]. It has shown promising efficacy in a subset of CCA patients [46]. However, the translational utility of PDX models in immunotherapy research has been limited by inherent biological disparities between humans and immunodeficient mice. To address these constraints, humanized mice engrafted with a human immune system have been developed, serving as a crucial tool for tumor immunotherapy [47, 48]. On the other hand, the PDO models can be co-cultured with peripheral blood mononuclear cells to replicate the in vivo immune microenvironment to predict the treatment response to immunotherapy [29]. Furthermore, the tumor microenvironment plays a fundamental role in driving the aggressiveness and therapeutic resistance of CCA [49, 50]. PDO models can be co-cultured with cancer-associated fibroblasts to simulate the tumor microenvironment and investigate interactions between tumor cells and their microenvironment [51, 52].

While our study models initial chemosensitivity, clinical treatment involves multiple cycles that can lead to acquired resistance, potentially diverging from PDO predictions. A critical future direction is to explore these resistance mechanisms. By generating resistant derivatives of PDO/PDX models through prolonged drug exposure and analyzing them with multi-omics approaches (such as WES and RNA-seq), we can systematically identify the underlying genomic and transcriptomic alterations. Elucidating this evolution is vital to explain treatment failure and to inform new combination strategies that overcome resistance.

To guide the clinical translation of our platform, future work will prioritize two pivotal areas. First, we will focus on technical optimization to enhance its scalability and feasibility, primarily by reducing the turnaround time of the PDO drug screen and improving the establishment success rate. Second, we will expand the therapeutic scope of the platform by incorporating high-throughput testing of targeted agents based on WES data and developing co-culture systems to evaluate immunotherapies. This evolution is essential to transform our platform into a comprehensive, clinically actionable tool for personalizing CCA therapy.

Our study also has several limitations. First, although our model establishment rates are comparable to those in the literature, approximately 35–44% of patient samples failed to generate PDO or PDX models. This could introduce a selection bias, as tumors that successfully engraft may possess more aggressive biological characteristics. Furthermore, another key limitation of this study is that the small cohort of models subjected to WES (*n* = 2) precludes a systematic correlation between genomic alterations and drug response phenotypes. While we established the platform’s genomic fidelity, future work on a larger scale is required to identify and validate druggable targets. Our immediate future directions will therefore focus on expanding WES to our entire cohort of PDOs/PDXs to identify candidate alterations. Finally, while we have characterized the morphological subtypes of our iCCA models, a further limitation is the lack of definitive molecular subtyping (small-duct vs. large-duct) based on comprehensive IHC profiling. Incorporating such analyses in future studies will be crucial to deepen the understanding of the pathological and clinical relevance of our models.

## Conclusions

In conclusion, we successfully established both PDO and PDX models of CCA. Drug sensitivity testing highlighted the potential of PDOs as an efficient in vitro preclinical platform for predicting chemotherapy response. Given the greater time and cost investments associated with PDX models, PDOs present a more practical solution for large-scale drug screening, whereas PDXs remain invaluable for subsequent in vivo validation of efficacy, as demonstrated in this study. Together, our findings support the integrated use of PDO and PDX models as a powerful strategy to inform clinical decision-making and advance personalized therapy for CCA patients.

## Supplementary Information

Additional file 1: Table S1. Postoperative clinical data of CCA patients.

Additional file 2: Table S2. Composition of CCA organoid medium.

Additional file 3: Table S3. Timeline of PDX models establish.

Additional file 4: Table S4. High-frequency tumor mutation gene analysis by WES.

Additional file 5: Table S5. The IC50 of drug screening to chemotherapeutics of CCA PDOs.

Additional file 6: Table S6. The AUC of drug screening to chemotherapeutics of CCA PDOs.

Additional file 7: Table S7. The clinical data from seven CCA patients who received adjuvant chemotherapy after surgery and had drug screening results for their derived organoids.

Additional file 8: Figure S1. The impact of chemotherapy drugs on cell viability was assessed using an organoid-formation assay. Purple square, resistant; bule square, intermediate sensitive; no square, sensitive. Scale bars, 50 μm.

## Data Availability

All data needed to evaluate the conclusions in the paper are present in the paper and/or the supplementary material. Raw data will be available upon request (contact email: 5020200824@nankai.edu.cn).

## Abbreviations

CCA: Cholangiocarcinoma
iCCA: Intrahepatic cholangiocarcinoma
pCCA: Perihilar cholangiocarcinoma
dCCA: Distal cholangiocarcinoma
5-FU: 5-Fluorouracil
PDX: Patient-derived tumor xenograft
PDO: Patient-derived tumor organoid
Nude: BALB/c-nu
WES: Whole-exome sequencing
CNVs: Copy number variations
IC50: Half maximal inhibitory concentration
AUC: Area under the curve
H&E: Hematoxylin-eosin
IHC: Immunohistology
ANOVA: Analysis of variance
GEMOX: Gemcitabine and oxaliplatin
GP: Gemcitabine and cisplatin
SNVs: Single nucleotide variant
Indels: Insertions and deletions
