# Supplementary figures and images for: Individualized drug screening in cholangiocarcinoma using organoid models and patient-derived tumor xenograft
Source: BMC Cancer. 2025 Dec 31;26:173. doi: 10.1186/s12885-025-15495-w (PMC12870517; doi:10.1186/s12885-025-15495-w)

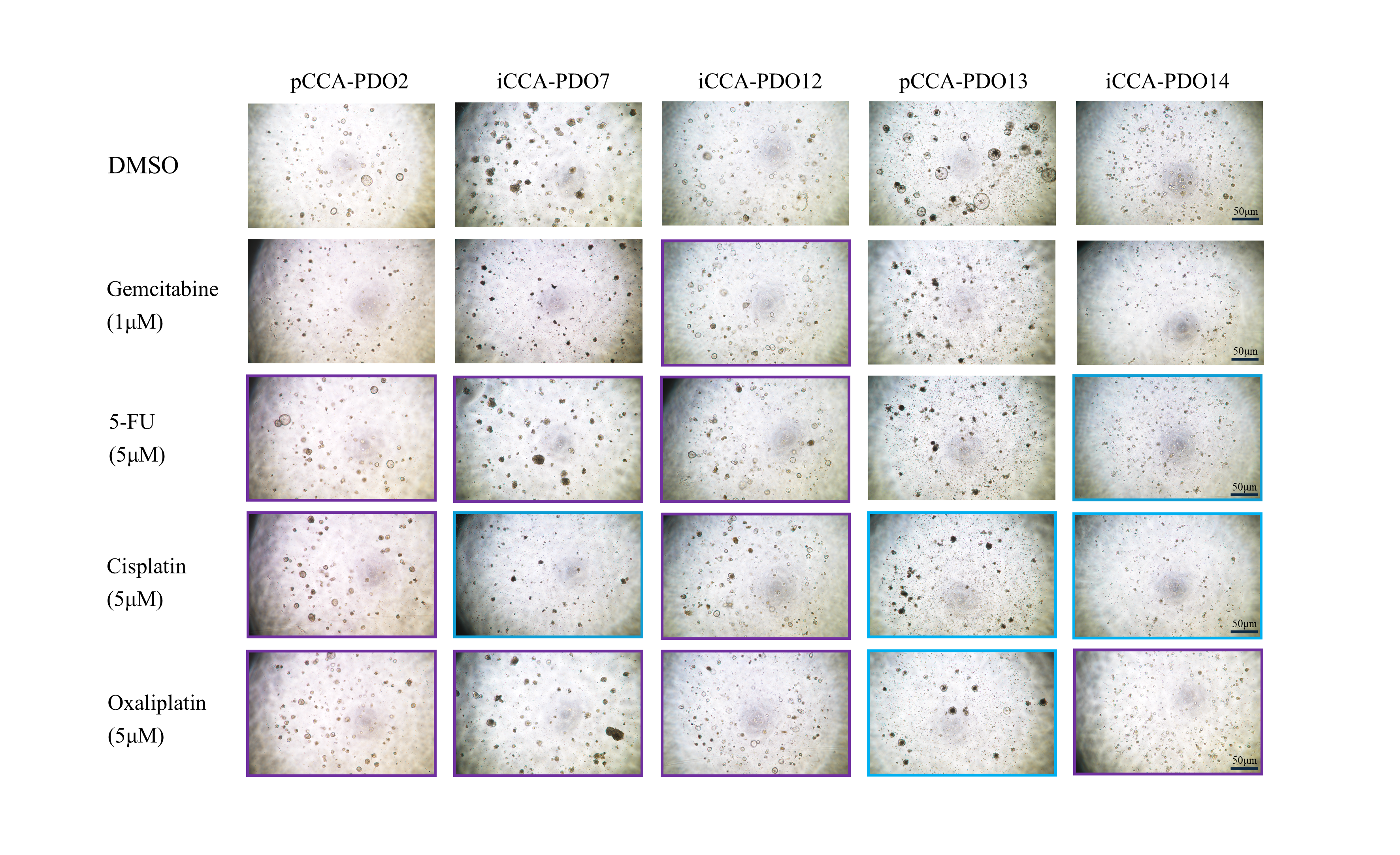

Supplement: Supplementary file 8 — Additional file 8: Figure S1. The impact of chemotherapy drugs on cell viability was assessed using an organoid-formation assay. Purple square, resistant; bule square, intermediate sensitive; no square, sensitive. Scale bars, 50 μm. [file 12885_2025_15495_MOESM8_ESM.tif]
